# Supplementary material for: SmRAV1, an AP2 and B3 Transcription Factor, Positively Regulates Eggplant’s Response to Salt Stress
Source: Plants (Basel). 2023 Dec 15;12(24):4174. doi: 10.3390/plants12244174 (PMC10747502; doi:10.3390/plants12244174)
Supplement: Supplementary file 1 [file plants-12-04174-s001.zip › Supplemental Table S1.pdf]

Table S1. Sequences of primer pairs were used in this study.

Primer name	Forward (5' to 3')	Reserves (5' to 3')	
SmRAV1	CAGATCAGCTCAGCCAGTGA	ATGATGCTGACTCCACTTCC	RT-qPCR
SmActin	CACTTAGCACCTTCCAGCAGATGT	GTACAACAGCAGACCTGAGTTCACT	RT-qPCR
SmGSTU10	GGGTGACCACTCTGCTTGAG	TGAAACGTAAAGCCATTTTGC	RT-qPCR
SmNCED1	GCCATCAACAAAAGAGTTTGG	TGGAGAATAGGAGGAGCTTGA	RT-qPCR
SmRAV1-	CTGTACAAGGGTACCCCCGGGATG	AGAGGATCCGTCGACCCCGGGTCAC	Subcellular
pBinGFP2	GACATAAGTTTCATAGATCAAGATCA	AAGGCATCAATTATAACCCT	localization
SmRAV1-	GGGGACAAGTTTGTACAAAAAAGCA	GGGGACCACTTTGTACAAGAAAGCT	VIGS
VIGS	GGCTTCCAAACGATGTACTGGAGCTG	GGGTCAGTACAAAAAGGAAAAGAAA	
