# Supplementary material for: SmRAV1, an AP2 and B3 Transcription Factor, Positively Regulates Eggplant’s Response to Salt Stress
Source: Plants (Basel). 2023 Dec 15;12(24):4174. doi: 10.3390/plants12244174 (PMC10747502; doi:10.3390/plants12244174)
Supplement: Supplementary file 1 [file plants-12-04174-s001.zip › Supplemental Table S2.pdf]

Table S2. Secondary structure of the SmRAV1 protein.

	Alpha helix (Hh)	Beta turn (Tt)	Extended strand (Ee)	Random coil (Cc)
SmRAV1	87 (23.77%)	18 (4.92%)	78 (21.31%)	183 (50.00%)
