# Supplementary figures and images for: SmRAV1, an AP2 and B3 Transcription Factor, Positively Regulates Eggplant’s Response to Salt Stress
Source: Plants (Basel). 2023 Dec 15;12(24):4174. doi: 10.3390/plants12244174 (PMC10747502; doi:10.3390/plants12244174)

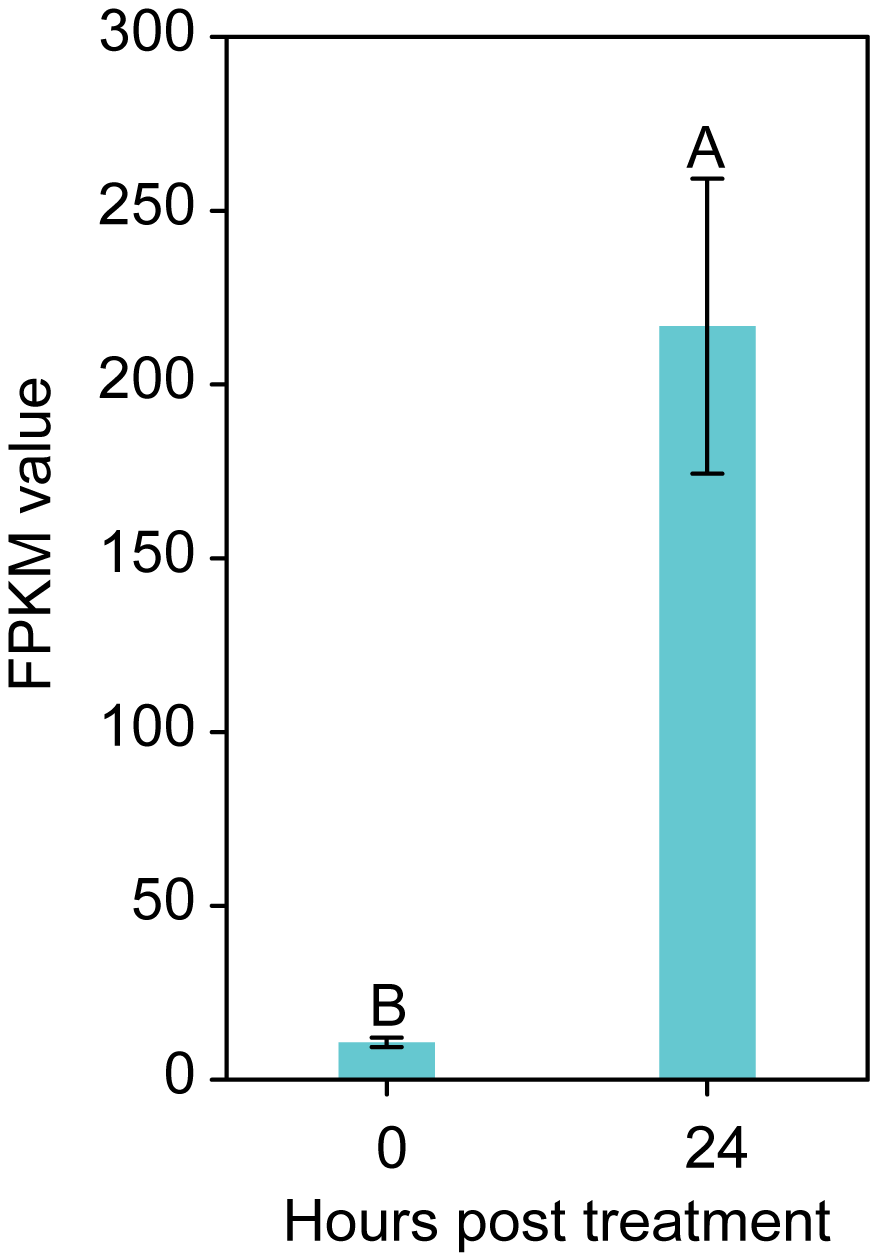

Supplement: Supplementary file 1 [file plants-12-04174-s001.zip › Figure S1.tif]

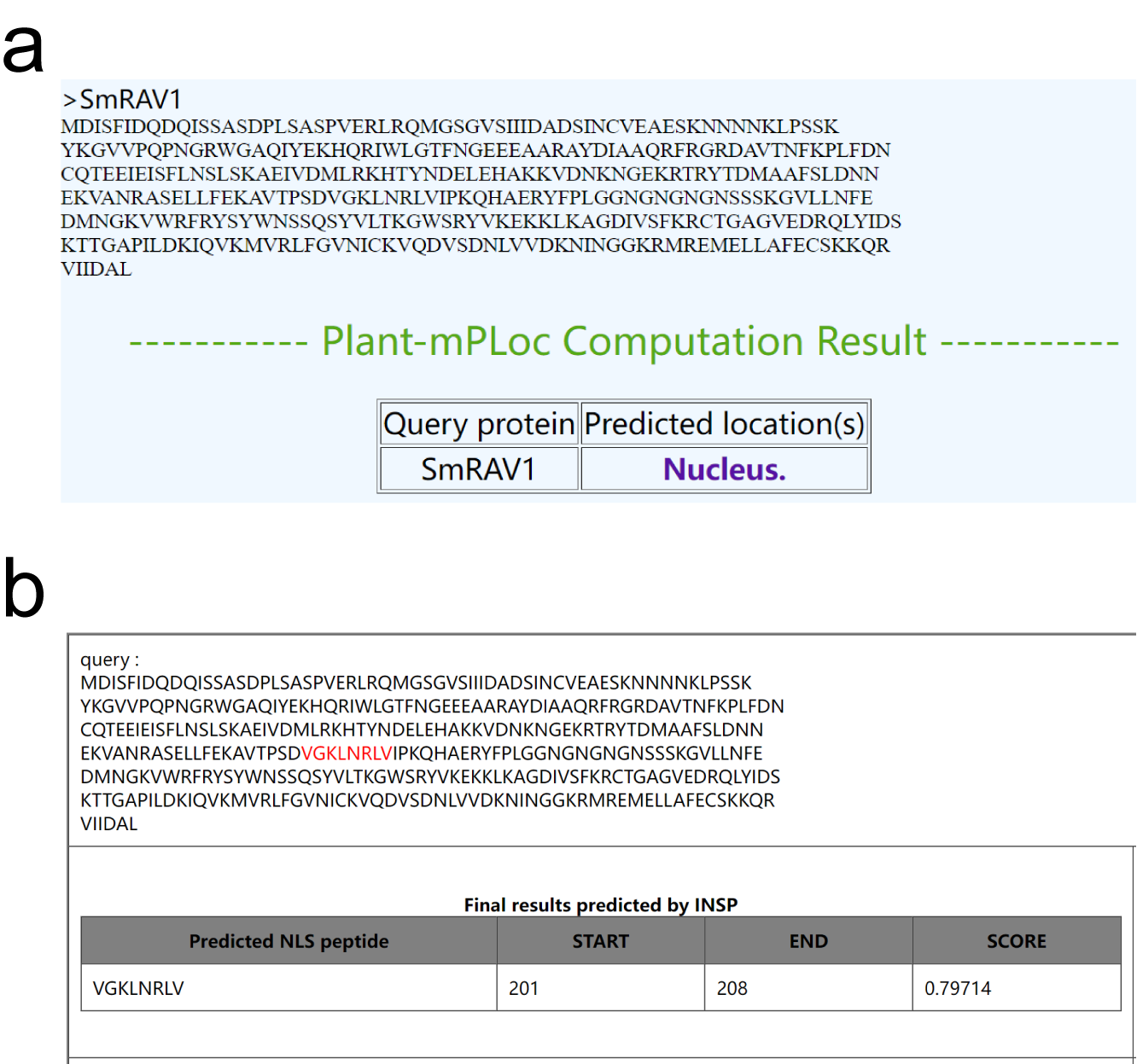

Supplement: Supplementary file 1 [file plants-12-04174-s001.zip › Figure S2.tif]
